# Genomic data resource of type strains of genus *Pseudoxanthomonas*

**DOI:** 10.1016/j.dib.2022.108145

**Published:** 2022-04-15

**Authors:** Kanika Bansal, Sanjeet Kumar, Prashant P. Patil, Shikha Sharma, Prabhu B. Patil

**Affiliations:** Bacterial Genomics and Evolution Laboratory, CSIR-Institute of Microbial Technology, Sector-39A, Chandigarh 160036, India

**Keywords:** *Pseudoxanthomonas*, Type strains, Phylogenomics, OrthoANI, Whole genome sequencing, Illumina MiSeq

## Abstract

Genus *Pseudoxanthomonas* represents a relatively newly characterized group of gamma-proteobacterium of environmental origin. Species of the genus have very similar morphology to strains belonging to *Xanthomonas, Xylella* and *Stenotrophomonas*. However, the genome resource of this genus was largely unexplored. The species belonging to the genus are from a wide range of environmental sites including hydrocarbon polluted fields. Here, we have provided the whole genome sequence of all available type strains of the genus of *Pseudoxanthomonas*. In order to deduce the differences with closely related genera, we have employed the whole genome-based investigation of the type species of genus *Pseudoxanthomonas*.

## Specifications Table

**Table:** 

Subject	Biological sciences
Specific subject area	Microbiology: Bacteriology
Type of data	Whole genome sequence assembled genome with gene annotation and phylogeny of genus *Pseudoxanthomonas* and its related genera.
How the data were acquired	Whole genome sequencing (WGS) library was prepared for Illumina MiSeq sequencing platform. Assembly of the raw reads were performed using SPAdes v3.10.
Data format	Raw Analyzed
Parameters for data collection	Sequencing library for all the available type strains were prepared for Illumina MiSeq following manufacturer's instructions. Sequencing was performed with 2*250 bp paired end sequencing kit.
Description of data collection	WGS data obtained from the sequencer was quality trimmed by control software of Illumina MiSeq. Raw reads were *de novo* assembled into high quality draft genome was performed using SPAdes v3.10 and quality checked using CheckM v1.1.0
Data source location	Institution: CSIR-Institute of Microbial Technology, Chandigarh City/Town/Region: Chandigarh Country: INDIA
Data accessibility	NCBI: MWIP00000000: https://www.ncbi.nlm.nih.gov/nuccore/MWIP00000000 NCBI: PDWO00000000: https://www.ncbi.nlm.nih.gov/nuccore/PDWO00000000 NCBI: PDWN00000000: https://www.ncbi.nlm.nih.gov/nuccore/PDWN00000000 NCBI: PDWT00000000: https://www.ncbi.nlm.nih.gov/nuccore/PDWT00000000 NCBI: PDWS00000000: https://www.ncbi.nlm.nih.gov/nuccore/PDWS00000000 NCBI: PDWW00000000: https://www.ncbi.nlm.nih.gov/nuccore/PDWW00000000 NCBI: PDWU00000000: https://www.ncbi.nlm.nih.gov/nuccore/PDWU00000000 NCBI: PDWR00000000: https://www.ncbi.nlm.nih.gov/nuccore/PDWR00000000 NCBI: PDWL00000000: https://www.ncbi.nlm.nih.gov/nuccore/PDWL00000000 NCBI: PDWQ00000000: https://www.ncbi.nlm.nih.gov/nuccore/PDWQ00000000 NCBI: PDWM00000000: https://www.ncbi.nlm.nih.gov/nuccore/PDWM00000000 NCBI: PDWP00000000: https://www.ncbi.nlm.nih.gov/nuccore/PDWP00000000 NCBI: PDWV00000000: https://www.ncbi.nlm.nih.gov/nuccore/PDWV00000000 NCBI: PDWK00000000: https://www.ncbi.nlm.nih.gov/nuccore/PDWK00000000 NCBI: QOVG00000000: https://www.ncbi.nlm.nih.gov/nuccore/QOVG00000000
Related research article	Bansal, K., Kumar, S., Kaur, A., Singh, A. & Patil, P. B. (2021) Deep phylo-taxono genomics reveals *Xylella* as a variant lineage of plant associated *Xanthomonas* and supports their taxonomic reunification along with *Stenotrophomonas* and *Pseudoxanthomonas*. https://doi.org/10.1016/j.ygeno.2021.09.021[1].

## Value of the Data

• Species of genus *Pseudoxanthomonas* are from contaminated sites such as: heavy metal, oil, hydrocarbons etc. Genome resource of strains from such extreme environmental conditions will aid in identification of genomic signatures underlying their bioremediation potential.
• These assembled genomes can be reused as a reference by the taxonomist and microbiologist in order to distinguish any putative species of the genera *Pseudoxanthomonas*.
• Present genome resource of type strains will be valuable in addressing the taxonomic ambiguities of the family *Lysobacteraceae* and order *Lysobacterales*.

## 1 Data Description

**Fig. 1:**
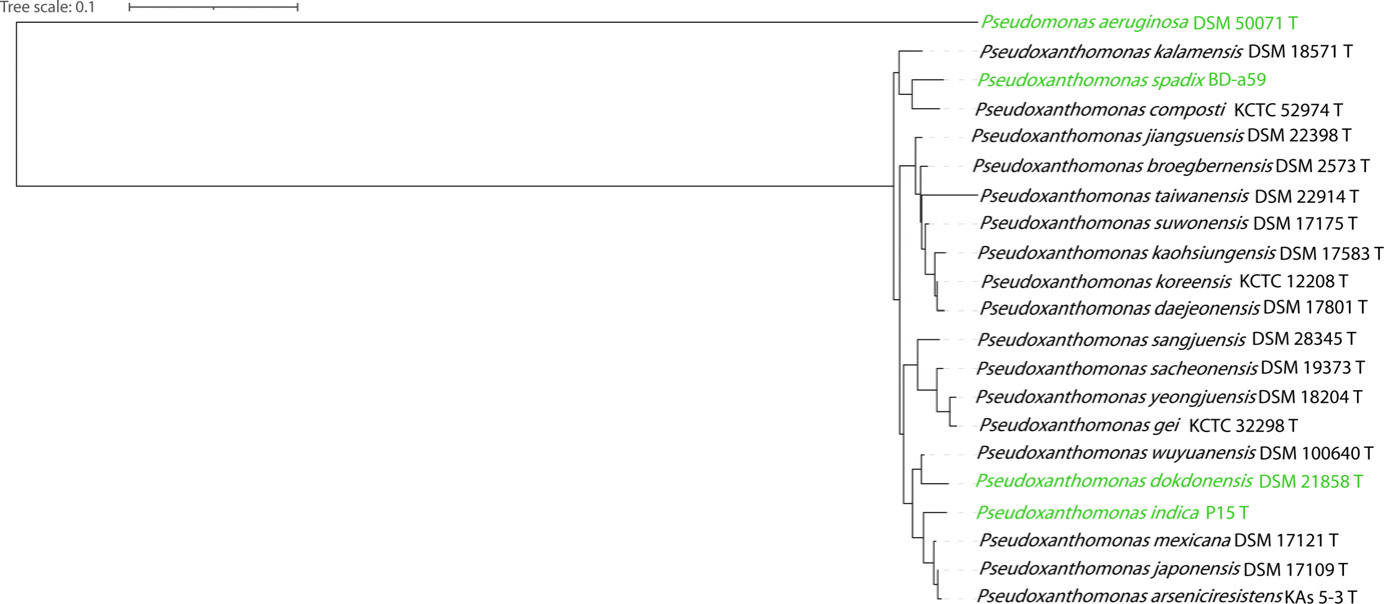
Phylogenetic tree construction with maximum-likelihood method based on the 16S rRNA gene sequence of different species of genus *Pseudoxanthomonas. Pseudomonas aeruginosa* DSM 50071^T^ was used as outgroup. Genomes sequenced in the present study are in black and genomes from public repository are in green color. Bootstrap values are shown at the node of each cluster in blue color as percentage of 1000 replicates.

Here, we have performed whole-genome sequencing of the 15 type strains of genus *Pseudoxanthomonas* comprising of 14 valid species and one non-valid type strain of *P. jiangsuensis* DSM 22398^T^ based on LPSN latest classification v2.0. Whole genome data of *P. dokdonesis* DSM 21858^T^, *P. indica* P15^T^ and *P. spadix* BD-a59 were obtained from the public repository of NCBI (Table 1). *P. helianthi* NRBC 110414^T^ [2] and *P. putridarboris* LMG 25968^T^ [3] could not retrieved and thus whole genome sequence information is not included in the study. 16S rRNA based phylogeny of all the twenty species of the genus *Pseudoxanthomonas* is depicted in Fig. 1. Whole genome sequence of the type strains of the genus *Pseudoxanthomonas* can be a valuable resource in taxonogenomics study of family *Lysobacteraceae* and its close relatives such as *Xanthomonas* and *Stenotrophomonas* [4,5]. Extreme environmental isolates such as *P. taiwanensis* [6] could be one of the key biotechnologically importance species to explore the heat stress mechanism. Genome resource of species of *P. broegbernensis, P. indica, P. kalamensis, P. kaohsiungensis, P. sacheonensis, P. spadix and P. jiangsuensis* [7], [8], [9], [10], [11], [12], [13] could be used for studying the stress tolerant genomics determinants.

**Table 1:** Genome assembly statistics of the species of genus *Pseudoxanthomas*.

Strain name	Genome size (bps)	Fold	# Contigs	N50 (bps)	% GC	Completeness/ Contamination	# CDS	tRNA + rRNA	#Putative Plasmids	Accession number	Refs.
*P. broegbernensis* DSM 12573^T^	3,547,767	267x	157	175,278	70.6	99.66/1.74	3024	54 + 3	6	MWIP00000000	Current study
*P. kaohsiungensis* DSM 17583^T^	3,774,556	269x	100	199,653	69.68	99.66/1.42	3420	52 + 3	6	PDWO00000000	Current study
*P. daejeonensis* DSM 17801^T^	3,563,566	131x	38	227,294	68.89	99.66/0.11	3143	58 + 3	3	PDWN00000000	Current study
*P. yeongjuensis* DSM 18204^T^	3,937,688	137x	28	610,842	65.11	99.95/0.76	3389	53 + 3	1	PDWT00000000	Current study
*P. sacheonensis* DSM 19373^T^	4,036,514	101x	44	165,077	64.3	100/0.91	3582	50 + 3	1	PDWS00000000	Current study
*P. japonensis* DSM 17109^T^	4,075,711	111x	74	108,688	67.3	99.95/0.34	3673	50 + 3	4	PDWW00000000	Current study
*P. wuyuanensis* DSM 100640^T^	4,686,433	112x	86	149,132	65.75	100/1.08	4003	51 + 6	6	PDWU00000000	Current study
*P. sangjuensis* DSM 28345^T^	3,289,016	91x	76	105,206	68.68	99.95/0.46	2908	51 + 3	3	PDWR00000000	Current study
*P. jiangsuensis* DSM 22398^T^	3,790,571	171x	154	121,587	70.35	99.31/1.03	3389	50 + 3	10	PDWL00000000	Current study
*P. kalamensis* DSM 18571^T^	3,034,522	239x	162	478,688	65.88	99.84/1.50	2697	48 + 3	-	PDWQ00000000	Current study
*P. koreensis* KCTC12208^T^	3,049,736	74x	93	60,801	70.17	99.48/0.04	2681	52 + 3	7	PDWM00000000	Current study
*P. suwonensis* DSM 17175^T^	3,428,455	65x	66	104,754	70.36	99.66/0.34	3071	55 + 3	2	PDWP00000000	Current study
*P. mexicana* DSM17121^T^	3,965,467	32x	115	63,754	67.4	99.49/1.71	3660	54 + 6	10	PDWV00000000	Current study
*P. taiwanensis* DSM22914^T^	3,043,352	69x	160	39,985	72.08	99.16/0.34	2729	56 + 3	14	PDWK00000000	Current study
*P. gei* KCTC32298^T^	3,431,103	173x	50	332,530	65.46	99.95/0.41	3,122	45 + 4	-	QOVG00000000	Current study
*P. dokdonesis* DSM21858^T^	3,553,658	170x	34	-	64.48	99.59/0.41	3153	50 + 2	NA	LDJL01000000	[4]
*P. indica* P15^T^	3,960,920	-	3	-	65.4	99.89/0.41	3593	49 + 6	NA	FUZV01000000	DOE-Joint Genome Institute
*P. spadix* BD-a59	3,452,554	-	1	-	67.65	97.1/1.3	3153	50 + 3	NA	CP003093	[22]

## 2 Experimental Design, Materials and Methods

### 2.1 Bacterial strains and culture conditions

Type strains of the genus *Pseudoxanthomonas* were procured from two culture collection of Korean Collection for Type Cultures (KCTC) and The Leibniz Institute DSMZ-German Collection of Microorganisms and Cell Cultures (Table 1). Ampoules containing respective bacterial cultures were processed in the recommended media and condition in accordance with the bacterial strains collection.

### 2.2 Genome sequencing, assembly and annotation

Bacterial genomic DNA was extracted using ZR Fungal/Bacterial DNA MiniPrep Kit (Zymo Research, Irvine, CA, USA) and quantified using Qubit 2.0 Fluorometer (Thermo Fisher Scientific, Waltham, MA, USA). 1 ng of DNA sample was used in the preparation of Illumina sequencing libraries using Nextera XT sample preparation kit with dual indexing following provider's instructions. Sequencing libraries were pooled and sequenced in-house on Illumina MiSeq platform with 2*250 bp paired-end sequencing kit.

The raw sequencing reads were assembled into the high-quality draft genome using SPAdes v3.10 [14] which is a de Bruijn graph-based assembler for the bacterial genome. Quality of the assembled genome was accessed using QUAST v4.4 [15] and overall coverage of the assembled genome was calculated using BBMap [16]. Presence of putative plasmid in the assembled genome was accessed using plasmidSPAdes [17] with a minimum cut-off of 1Kb length. The assembled genomes were annotated using the NCBI prokaryotic genome annotation pipeline [18]. Assembly information with the putative number of plasmids is summarized in Table 1.

### 2.3 Phylogenetic assessment

Phylogenetic analysis based on the traditional 16S rRNA gene sequence was performed, for which 16S rRNA gene sequence was fetched from the respective assembled genome using from a standalone academic version of RNAmmer v1.2 [19] except for the type strains for species *P. spadix, P. helanthi* and *P. putridarboris.* 16S rRNA for these 3 species were taken from LPSN of the respective species definition. Multiple sequence alignment of 16S rRNA gene sequences was performed using ClustalW [20]. Phylogenetic tree based on Maximum Likelihood method with 1000 bootstrap replication was generated using MEGA v7.0.18 [21].

## Ethics Statement

There is no ethical concern involved in the study.

## Author Contributions

SK, SS and PPP have carried out strain procurement from culture collection and strain revival. KB and SK have performed whole genome sequencing and submission of assembled genomes to NCBI. PBP has conceived the study and participated in the design. All the authors have read and approved the manuscript.

## Declaration of Competing Interest

The authors declare that they have no known competing financial interests or personal relationships which have or could be perceived to have influenced the work reported in this article.

## Data Availability

Genomic data resource of type strains of genus *Pseudoxanthomonas* (Original data) (NCBI genome).

## References

[1] Bansal K. (2021). Deep phylo-taxono genomics reveals *Xylella* as a variant lineage of plant associated *Xanthomonas* and supports their taxonomic reunification along with *Stenotrophomonas* and *Pseudoxanthomonas*. Genomics.

[2] Kittiwongwattana C., Thawai C. (2016). *Pseudoxanthomonas helianthi* sp. nov., isolated from roots of Jerusalem artichoke (Helianthus tuberosus). Int. J. Syst. Evol. Microbiol..

[3] Lee J.K. (2017). *Pseudoxanthomonas putridarboris* sp. nov. isolated from rotten tree. Int. J. Syst. Evol. Microbiol..

[4] Patil P.P. (2016). Genome sequence of type strains of genus *Stenotrophomonas*. Front. Microbiol..

[5] Kumar S. (2019). Phylogenomics insights into order and families of *Lysobacterales*. Access Microbiol..

[6] Chen M.Y. (2002). *Pseudoxanthomonas taiwanensis* sp. nov., a novel thermophilic, N_2_O-producing species isolated from hot springs. Int. J. Syst. Evol. Microbiol..

[7] Finkmann W. (2000). Characterization of N_2_O-producing *Xanthomonas*-like isolates from biofilters as *Stenotrophomonas nitritireducens* sp. nov., *Luteimonas mephitis* gen. nov., sp. nov. and *Pseudoxanthomonas broegbernensis* gen. nov., sp. nov. Int. J. Syst. Evol. Microbiol..

[8] Kumari K. (2011). *Pseudoxanthomonas indica* sp. nov., isolated from a hexachlorocyclohexane dumpsite. Int. J. Syst. Evol. Microbiol..

[9] Harada R.M., Campbell S., Li Q.X. (2006). *Pseudoxanthomonas kalamensis* sp. nov., a novel gammaproteobacterium isolated from Johnston Atoll, North Pacific Ocean. Int. J. Syst. Evol. Microbiol..

[10] Chang J.S. (2005). *Pseudoxanthomonas kaohsiungensis*, sp. nov., a novel bacterium isolated from oil-polluted site produces extracellular surface activity. Syst. Appl. Microbiol..

[11] Lee D.S. (2008). *Pseudoxanthomonas sacheonensis* sp. nov., isolated from BTEX-contaminated soil in Korea, transfer of *Stenotrophomonas dokdonensis* Yoon et al. (2006) to the genus *Pseudoxanthomonas* as *Pseudoxanthomonas* dokdonensis comb. nov. and emended description of the genus *Pseudoxanthomonas*. Int. J. Syst. Evol. Microbiol..

[12] Young C.C. (2007). *Pseudoxanthomonas spadix* sp. nov., isolated from oil-contaminated soil. Int. J. Syst. Evol. Microbiol..

[13] Wang G.L. (2011). *Pseudoxanthomonas jiangsuensis* sp. nov., a DDT-degrading bacterium isolated from a long-term DDT-polluted soil. Curr. Microbiol..

[14] Bankevich A. (2012). SPAdes: a new genome assembly algorithm and its applications to single-cell sequencing. J. Comput. Biol..

[15] Gurevich A. (2013). QUAST: quality assessment tool for genome assemblies. Bioinformatics.

[16] Bushnell, B., BBMap: a fast, accurate, splice-aware aligner. (2014). (No. LBNL-7065E). Lawrence Berkeley National Lab.(LBNL), Berkeley, CA (United States) doi: https://www.osti.gov/servlets/purl/1241166.

[17] Antipov D. (2016). PlasmidSPAdes: assembling plasmids from whole genome sequencing data. Bioinformatics.

[18] Tatusova T. (2016). NCBI prokaryotic genome annotation pipeline. Nucleic Acids Res..

[19] Lagesen K. (2007). RNAmmer: consistent and rapid annotation of ribosomal RNA genes. Nucleic Acids Res..

[20] Larkin M.A. (2007). Clustal W and Clustal X version 2.0. Bioinformatics.

[21] Kumar S., Stecher G., Tamura K. (2016). MEGA7: molecular evolutionary genetics analysis version 7.0 for bigger datasets. Mol. Biol. Evol..

[22] Lee S.H. (2012). Complete genome sequence of the BTEX-degrading bacterium *Pseudoxanthomonas spadix* BD-a59. J. Bacteriol..

